# Biosynthesis and Characterization of Silver Nanoparticles Produced by *Phormidium ambiguum* and *Desertifilum tharense* Cyanobacteria

**DOI:** 10.1155/2022/9072508

**Published:** 2022-02-28

**Authors:** Amira L. Hanna, Hayam M. Hamouda, Hanan A. Goda, Mahmoud W. Sadik, Farahat S. Moghanm, Adel M. Ghoneim, Muneefah A. Alenezi, Sultan F. Alnomasy, Pravej Alam, Tarek R. Elsayed

**Affiliations:** ^1^Microbiology Department, Division of Basic Medical Science, Egyptian Drug Authority EDA (National Organization for Drug Control and Research NODCAR), Giza 12553, Egypt; ^2^Department of Microbiology, Faculty of Agriculture, Cairo University, Giza 12613, Egypt; ^3^Department of Environmental Biotechnology, College of Biotechnology, Misr University for Science and Technology, Giza, Egypt; ^4^Soil and Water Department, Faculty of Agriculture, Kafrelsheikh University, Kafr El-Sheikh 33516, Egypt; ^5^Agricultural Research Center, Field Crops Research Institute, Giza 12112, Egypt; ^6^Biology Department, Faculty of Science, Tabuk University, Tabuk 71491, Saudi Arabia; ^7^Medical Laboratories Department, College of Applied Medical Sciences, Al- Quwayiyah, Shaqra University, Riyadh, Saudi Arabia; ^8^Department of Biology, College of Science and Humanities, Prince Sattam Bin Abdulaziz University, Al-Kharj 1942, Saudi Arabia

## Abstract

The world faces a challenge with the pervasion of multidrug-resistant bacteria that encourages scientists to develop and discover alternative, ecofriendly, and easy-to-produce new antibacterial agents. Our work is part of the greater effort of scientists around the world to achieve this goal by the biological synthesis of silver nanoparticles using cyanobacterial extracellular and intracellular components as nonchemical reducing agents. Two Egyptian cyanobacteria were isolated and identified according to 16S rRNA gene sequencing as *Phormidium ambiguum* and a novel species *Desertifilum tharense*. The sequences were deposited with accession numbers MW762709 and MW762710 for *Desertifilum tharense* and *Phormidium ambiguum*, respectively, in the GenBank. The results of UV-Vis analysis showed promising extracellular Ag-NPs synthesis by *Desertifilum tharense* and *Phormidium ambiguum* under light conditions. Therefore, these Ag-NPs were characterized and evaluated for antibacterial and antioxidant activity. TEM and SEM analyses revealed the spherical crystals with face-centered cubic structures and size range of 6.24–11.4 nm and 6.46–12.2 nm for Ag-NPs of *Desertifilum tharense* and *Phormidium ambiguum*, respectively. XRD and EDX results confirmed the successful synthesis of Ag-NPs in their oxide form or chloride form. The FTIR spectrum data confirmed the presence of hydroxyl and amide groups. *Desertifilum tharense* Ag-NPs displayed the largest inhibition zone that ranged from 9 mm against *Micrococcus luteus* ATCC 10240 to 25 mm against methicillin-resistant *Staphylococcus aureus* (MRSA) ATCC 43300. For *Phormidium ambiguum* Ag-NPs, the inhibition zone diameter was in the range of 9 mm to 18 mm. The biosynthesized Ag-NPs significantly inhibited the growth of medically important resistance-pathogenic Gram-positive and Gram-negative bacteria. The Ag-NPs of *Phormidium ambiguum* exhibited the highest scavenging activity of 48.7% when compared with that of *Desertifilum tharense*, which displayed 43.753%.

## 1. Introduction

Nanotechnology is a strong area of science, engineering, and technology. Concerning the design and manufacture of materials at the nanoscale, the products of nanotechnology are nanomaterials or nanoparticles (NPs) with dimensions less than 100 nm. Small sizes and large surface area-to-volume ratios of nanoparticles exhibit a larger surface-to-volume ratio, which is an influential feature to improve the physical, chemical, and biological properties of the original material. These properties include catalytic reactivity, thermal conductivity, antimicrobial activity, chemical steadiness, etc. According to the effectual crystallographic and physicochemical properties of NPs, the nanoparticles have substantial applications in industrial, medical, and biotechnological sectors [1–3].

Generally, the nanoparticles could be synthesized by applying different chemical and physical methods. The chemical methods include precipitation, solvothermal/hydrothermal, and sol-gel methods using different reducing and stabilizing agents. These processes are not environmentally friendly since they include the use of solvents, reducing, and stabilizing agents, which result in the release of significant amounts of hazardous and toxic materials in the form of nonbiodegradable compounds [4–7]. In physical biosynthesis, attrition and pyrolysis were applied to produce oxidized NPs. The physical methods are very expensive, labor-intensive, and time-consuming.

NPs in general, and silver-NPs in particular, are being employed in health, wound dressings, medicinal supplements, and industrial items for human use to suppress pathogen growth, as well as in cosmetics as an antiseptic, and in medical textiles, to eradicate microorganisms from the clinical environment proving the critical need for natural, environmentally acceptable, dependable, and safe nanoparticle production methods. Therefore, the biosynthesis of nanoparticles using different biological sources, such as plants, bacteria, fungi, and algae has great attention. The biological methods are cost-effective, nontoxic, pollutant-free, and ecofriendly methods [5, 9–14]. In recent decades, researchers were attracted toward cyanobacterial components that are potential low-cost biological reagents for silver nanoparticle biosynthesis.

Cyanobacteria, or blue-green algae, have attracted a lot of scientific attention for their ability to produce nanoparticles, not only because of their large biomass productivity, but also because of their ability to bioremediate hazardous metals and subsequently convert them to more manageable forms. Cyanobacteria can produce different organic (quaternary ammonium compounds, N-halamine compounds, chitosan, etc.) and inorganic metal and metal oxide NPs (gold, silver, platinum, zinc oxide, copper oxide, aluminum oxide, etc.).

The cyanobacterial synthesis of NPs is accomplished extracellularly or intracellularly. Extracellular biosynthesis involves the secretion of extracellular substances to mediate through electrostatic interactions or the production of extracellular reductase enzymes. Alternatively, the intracellular production of NPs associates with the activity of reductase enzymes and substance exchange processes [15, 16].

The physicochemical properties of silver nanoparticles (Ag-NPs) and their use extensively in the medical sector as antimicrobial and anticancer agents are the foremost reasons for considerable attention for their production. Silver nanoparticles display efficient antibacterial activity through multiple mechanisms, including the formation of free radicals, thereby stimulating membrane damage [17], increasing the cell membrane permeability by the formation of holes or channels through it [18], and the disruption of cell proteins by binding with thiol and amino groups [19].

Based on the promising features and applications of silver nanoparticles, the intended objective of the present study was the screening of some Egyptian cyanobacterial isolates as model biological systems to synthesize the Ag-NPs. Furthermore, the physical properties and potential applications of Ag-NPs as antibacterial and antioxidant agents were evaluated.

## 2. Materials and Methods

### 2.1. Green Earth Crust Samples Collection

Two natural Earth crust samples were collected from Giza Governorate, El-Haraneya, near the ring road in locations between the longitude of 31° 10′ 15″ and latitude of 29° 58′ 20″ for the first sample, and the longitude of 31° 9′ 59″ and latitude of 29° 57′ 57″ for the second sample.

### 2.2. Cyanobacteria Isolation and Purification

The enrichment culture technique was applied to isolate cyanobacteria. 1gm of each green Earth crust sample was dispersed and aseptically added to 25 ml of liquid BG-11 medium [20] inside a horizontal falcon tube. The inoculated medium was incubated under continuous illumination using Philips Fluorescent white lamps at a relatively low light intensity (400–500 lux) at 30 °C for ten days.

Different methods are used to obtain cyanobacteria monocultures (pure culture), depending on the cyanobacteria shape (coccoid or filamentous), cell size, and the degree of motility. A pure culture is not always possible because cyanobacteria may have such relationships with other microorganisms.

The single filament technique was used to obtain axenic cultures of filamentous cyanobacteria. Whole trichomes or hormogonia will glide on the wall of the tube for a few days. This technique was applied by picking these individual migrating trichomes using a Pasteur pipette, and then the single filament was cleaned with the sterile liquid medium in a Petri dish. For more purification, firstly, the motile filamentous cyanobacteria were dragged inside the BG-11 agar medium, allowing them to glide away. Then, remove any attached contaminations as described by Waterbury [21]. Some rapidly gliding filaments can clean themselves from any bacterial contaminants within hours of inoculum. Then, the purified isolates were transferred in flasks containing 250 ml of liquid BG-11 (pH 7). All pure isolates were maintained under photoautotrophic growth conditions as aforementioned.

### 2.3. Morphological Characterization of Cyanobacteria

The pure isolates were characterized by the preparation of a wet portion that was examined microscopically [20]. The filamentous nature, size, shape of vegetative cells, presence of heterocyst, and akinetes were identified.

### 2.4. Molecular Identification of Cyanobacteria

The cyanobacterial isolates were identified according to 16S rRNA sequencing. The isolates were cultivated in BG-11 broth for 30 days. After centrifugation of the broth culture at 6000 g for 10 min, the harvested biomass was washed three times using 0.85% NaCl saline solution, and genomic DNA was extracted using GeneJET Genomic DNA purification Kit (ThermoFisher Scientific, Republic of Lithuania). The purity of the extracted DNA was checked using both UV-Vis NanoDrop spectrophotometer (NanoDrop 2000, ThermoFisher Scientific, Germany) and agarose gel electrophoresis (Bio-rad, USA).

The 16S rRNA gene fragments were amplified using the cyanobacteria-specific primers CYA106 F (5′-CGGACGGGTGAGTAACGCGTGA-3′) and CYA781 R (5′-GACTACWGGGGTATCTAATCCCWTT-3′) [22]. The amplification step was performed using Thermal cycler PCR (Bio-Rad T100, USA). The PCR conditions for the 16S rRNA gene were as follows: initial denaturation step at 95°C for 12 min, followed by 30 cycles of 94°C for 1 min, 56°C for 1 min, and 72°C for 2 min, and one extension step at 72°C for 10 min. The PCR products were checked via agarose gel electrophoresis, purified using a gel extraction kit, and sequenced by Macrogen, Inc., Seoul, South Korea, by an automatic ABI 370×1 DNA Sequencer (Applied Biosystem, USA). The sequences were analyzed by applying the BLAST V2.0 software (http://www.ncbi.nlm.nih.gov/BLAST/).

### 2.5. Phylogenic Analysis of Cyanobacteria Isolates

The Neighbor-Joining method is used to conclude the evolutionary history [23]. The tree computes using the maximum composite likelihood method [24]. The analysis involves 13 nucleotide sequences, in which two sequences of 16S rRNA gene are amplified from cyanobacterial isolates, however, 11 sequences representing the most similar hits are obtained from the NCBI gene bank database. Evolutionary analyses were conducted by the MEGA5 software.

### 2.6. Biosynthesis of Silver Nanoparticles (Ag-NPs) by Cyanobacteria

Cyanobacteria isolates were tested for their ability to create Ag-NPs from extracellular metabolites (cyanobacteria culture supernatant) as well as intracellular metabolites (after exploding the cell and the internal metabolites come out) both in the light and in the dark. According to the method of Patel *et al.* [2], after 15 days of incubation, the broth with cyanobacterial cultures was centrifuged (Universal 16r, Hettich) at 6000 rpm/10 min at 10 °C.

For the extracellular synthesis of Ag-NPs, the supernatant was added to the AgNO_3_ solution to reach the final concentration of 1 mM AgNO_3_. To evaluate the synthesis of Ag-NPs intracellularly, the harvested biomass was washed at least twice with sterile distilled water, kept at −20°C overnight to facilitate the complete lysis of the cells [25], and then 0.5 Gram of wet weight biomass was suspended in 10 ml of 1 mM AgNO_3_ solution, pH 7. In both experiments, incubation was done at 30 °C/48 h, either under direct light provided by fluorescent-white lamps or in dark conditions provided by wrapping the tubes with aluminum foil. As a control, the AgNO_3_ solution, supernatant, and 0.5 Gram of wet weight biomass were suspended in 10 ml of sterile water and incubated under the same conditions.

### 2.7. Characterization of Ag-NPs

Dried nanoparticles are used to apply some characterization tests. To dry the Ag-NPs synthesized by isolated cyanobacteria, the Ag-NPs suspension obtained after incubation for 72h was centrifuged at 6000 rpm for 15 min at 10°C. The pellet was washed three times with sterile distilled water, spread in Petri plates, and dried at 30°C for 24 h. The dried nanoparticles were scraped by a scalpel, harvested, weighed, and stored in a sterile microtube for quantitative estimation [26].

The following characterization tests were applied to confirm the formation of Ag-NPs by isolated cyanobacteria.

#### 2.7.1. Visual Color Change Test

The formation of silver nanoparticles was detected by the visual color change from pale yellow to brown within the incubation time.

#### 2.7.2. Ultraviolet-Visible (UV–Vis) Spectroscopic Analysis

In time intervals of 1 and 24 h, 1 ml from each sample was centrifuged at 6000 rpm for 5 min. The absorption measurements in the wavelength range of 300 nm to 550 nm were estimated using UV-Vis spectroscopy (Specord 210 plus, Analytic Jena, Germany). The isolates that showed an absorption peak in the range between 400 and 450 nm were considered silver nanoparticle-producing cyanobacteria [27].

#### 2.7.3. Scanning Electron Microscopy (SEM)

After 48h, SEM was used to characterize the surface morphology of nanoparticles at an accelerating voltage of 30 kV. After centrifuging the Ag-NPs solution, the precipitate was allowed to dry. The dried nanoparticles were coated with gold by a coater to prevent building-up the electrical charges [12]. Also, Ag-NPs characterize in a particle suspension.

#### 2.7.4. Transmission Electron Microscopy (TEM)

TEM gives a 1,000-fold higher morphological resolution of the size and shape compared to SEM [27]. Two-dimensional and high-resolution Ag-NPs images were captured with TEM (Jeol, JEM^−1^400, Japan). A drop of Ag-NPs suspension was placed on copper-grid carbon coated with 300 mesh palladium and carbon and allowed to dry. The Ag-NPs morphology was observed by TEM processed at an operating voltage of 80 kV.

#### 2.7.5. X-Ray Diffraction (XRD) Analysis

The crystal structure of Ag-NPs was characterized using an X-ray-dx (pert pro PANalytical diffractometer). The powdered Ag-NPs were penetrated by X-rays and scanned across the region of 2*θ*, from the range of 0° to 80°.

#### 2.7.6. Energy Dispersive X-Ray (EDX)

EDX was used to determine the elemental composition of metal nanoparticles. Energy-dispersive X-ray spectroscopy uses the natural advantages of light photons. Generally, EDX operates by recording X-ray signals, energy, and intensity distribution generated using a focused electron beam [27, 28]. Analysis was processed with EDAX FEI INSPECT instrument across layout: kV: 25.00; take-off: 34.58; tilt: 0.00; AMPT: 102.4; resolution: 129.66; detector type: SUTW-sapphire; EDAX ZAF quantification standardless sec.

#### 2.7.7. Fourier Transform Infrared Spectroscopy (FTIR) Analysis

The chemistry and variations of the functional groups attached to Ag-NPs surface are identified with an FTIR spectrometer (NICOLET 380, China). The dried nanoparticles mix with potassium bromide in a ratio of 1 : 100. The sample of 100 *μ*l was placed in the attenuated total reflectance (BRUKER) analyzer. The silver nanoparticle solution was analyzed by ATR-FT-IR. The IR-rays spectrum was scanned across 4000–400/cm, with diffuse reflectance mode (DRS-800) within 4 cm^−1^ resolution [27] (https://www IR Spectrum Table and Chart, Sigma-Aldrich).

### 2.8. Antibacterial Efficiency of Ag-NPs

The antibacterial activity of Ag-NPs synthesized by isolated cyanobacteria was applied by well diffusion assay [29] against six bacterial strains, including Gram-positive and Gram-negative bacteria. These strains were *Bacillus cereus* ATCC 10876, Methicillin-resistant *Staphylococcus aureus* (MRSA) ATCC 43300, *Micrococcus luteus* ATCC 10240, *Pseudomonas aeruginosa* ATCC 9027, *Salmonella typhimurium* ATCC 14028, and *Escherichia coli* O157 : H7 wild type strain 93111 as a representative enterohaemorrhagic *E*. *coli* (EHEC). The broth culture of each bacterial strain was prepared in soybean casein digest broth (OXOID) with incubation at the optimum temperature for 24h. The optical density (OD600) of each culture was adjusted to 0.5 ± 0.1 using the UV/Vis spectrophotometer. Each culture was spread on the surface of nutrient agar using sterile cotton swabs, in which the wells of 10 mm diameter were prepared using a sterile cork borer. Each well was filled with 100 *μ*l from vancomycin hydrochloride (100 mg/ml) as a positive control, 100 *μ*l Ag-NPs resulting solution (1 mM), or AgNO3 solution (1 mM). The plates were incubated for 24 h at the optimum temperature for each microbe. The inhibitory effect of Ag-NPs was estimated by measuring the inhibition zone diameter. Also, the relative inhibition percentage was calculated according to the following formula of [30]:

(1) $$\begin{array}{c} \text{Relative percentage inhibition of AgNP}s=\;\frac{100\left(x-y\right)}{z-y\;} \end{array}$$

x: total inhibition area of Ag-NPs, y: total inhibition area of AgNO_3_ solution 1 mM, and z: total inhibition area of the standard drug.

### 2.9. Antioxidant Activity of Ag-NPs

The capability of cyanobacterial Ag-NPs to scavenge 2, 2-diphenyl^−1^-picrylhydrazyl (DPPH) radicals was assessed by mixing 100 *μ*l of Ag-NPs solution in 1 ml of 0.1 mM DPPH methanolic-solution. The mixture was vortexed and incubated at 37°C for 30 min in the dark. The absorbance of the samples was measured at 515 nm. The free radical scavenging activity was calculated using the following equation:

(2) $$\begin{array}{c} \text{DPPH scavenging activity }\left(\text{\%}\right)\left(\text{antioxidant activity \%}\right)=\;\frac{\left(A0-A1\right)}{A0}\;\times \;100 \end{array}$$

where *A*_0_ is the absorbance of the control reaction, and *A*_1_ is the absorbance of the reaction mixture [31].

## 3. Results and Discussion

### 3.1. Cyanobacteria Identification by Light Microscope Examination

**Figure 1:**
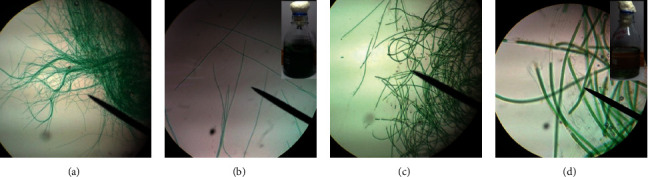
Light micrographs of cyanobacterial isolates AM5E—(a) 10x and (b) 40x—and AM6E—(c) 10x and (d) 40x.

Two isolates of cyanobacteria (AM5E and AM6E) were purified and characterized morphologically according to Rippka et al. [20]. The AM5E and AM6E isolates were characterized as nonheterocystous cyanobacteria filamentous (Figures 1(a)–1(d)). Also, the filaments were unbranched. No gas vacuoles were observed within the cells. The filaments of AM5E were long, thin, straight, or slightly curvy, and they were nonconstructed. Also, they were motile and had sharp apical terminals. The filaments may exist in a separate state or bundles. The filaments of AM6E isolate were thick and separating (Figure 1(c) and d). The filaments were slightly motile and had round ends.

### 3.2. Molecular Identification and Phylogenetic Analysis of Cyanobacterial Isolates

**Figure 2:**
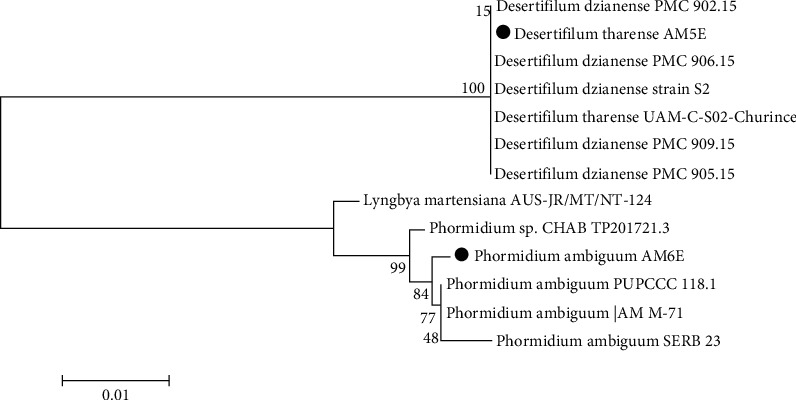
A neighbor-joining phylogenetic tree (dark circles) with the closest hits obtained from the NCBI gene bank.

According to the 16S rRNA gene sequence of the two isolates, AM5E showed 100% similarity to *Desertifilum tharense* and *Desertifilum dzianese*, while AM6E showed 99.6% similarity to *Phormidium ambiguum*. The phylogenetic relatedness was confirmed in the neighbor-joining tree (Figure 2). Sequences were deposited in the NCBI GenBank as *Desertifilum tharense* AM5E (accession number: MW762709) and *Phormidium ambiguum* AM6E (accession number: MW762710).

### 3.3. Characterization of Biosynthesized Silver Nanoparticles (Ag-NPs) by Cyanobacteria

#### 3.3.1. Visual Color Change

**Figure 3:**
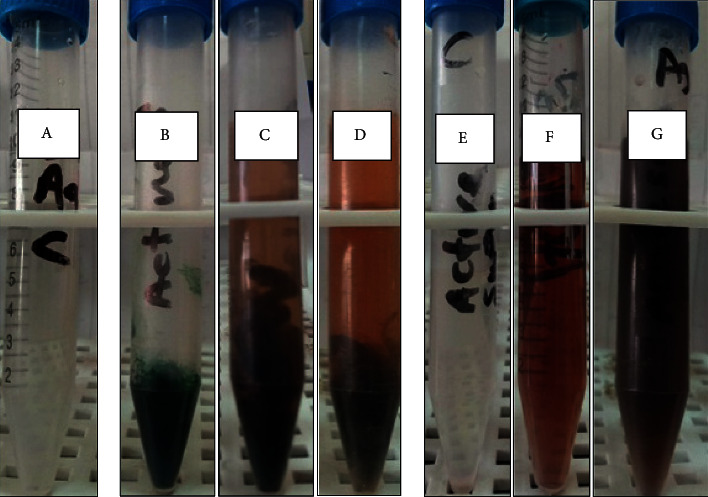
Color change during the reduction process of silver Ag-NPs extracellularly and intracellularly using *Desertifilum tharense*. (a) 1 mM silver nitrate solution (control 1). (b) Cell suspension in water (control 2). (c) The formation of Ag-NPs intracellularly under dark conditions after 48 h. (d) The formation of Ag-NPs intracellularly under light conditions after 48 h. (e) Cell-free supernatant (control 3). (f) The formation of Ag-NPs extracellularly under dark conditions after 48 h. (g) The formation of Ag-NPs extracellularly under light conditions after 48 h.

**Figure 4:**
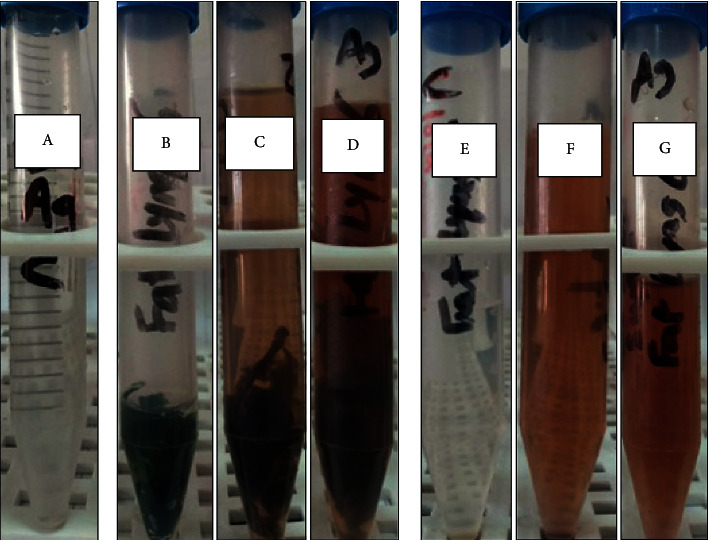
Color change during the reduction process of silver Ag-NPs extracellularly and intracellularly using *Phormidium ambiguum*. (a) 1 mM silver nitrate solution (control 1). (b) Cell suspension in water (control 2). (c) The formation of Ag-NPs intracellularly under dark conditions after 48 h. (d) The formation of Ag-NPs intracellularly under light conditions after 48 h. (e) Cell-free supernatant (control 3). (f) The formation of Ag-NPs extracellularly under dark conditions after 48 h. (g) The formation of Ag-NPs extracellularly under light conditions after 48 h.

In this study, the brown color observed after 1 hr, either for *Desertifilum tharense* (Figure 3) or *Phormidium ambiguum* (Figure 4), indicated their ability to synthesize Ag-NPs extra- and intracellularly under both light and dark conditions. For both isolates, after 48 hours, the darkest brown color was perceived under light conditions for extra- and intracellularly produced Ag-NPs. Comparing the intra- and extracellular biosynthesis of Ag-NPs, the dark brown color was evidence for promising extracellular biosynthesis by *Desertifilum tharense* or *Phormidium ambiguum*. Therefore, furthers characterization tests, antibacterial and antioxidant activities were assessed for Ag-NPs synthesized extracellularly under light conditions.

The reduction of silver atoms into silver nanoparticles using isolated cyanobacteria could be observed visually through the color change from pale yellow to brown. Generally, this process is time-dependent as the intensity of the brown color is directly proportional to reaction time [32]. Saifuddin *et al*. [33] reported that the color-changing was due to the excitation of surface plasmon vibrations in the resultant nanoparticle. The Ag-NPs produced by cyanobacteria were observed as highly stable for a variant time ranging from 1 to 120 h [34].

#### 3.3.2. UV–Vis Spectrophotometer Analysis

The formation of Ag-NPs detected by the visual observation of color change is confirmed by sharp peaks given in the visible region from the UV-Vis spectrum. Ag-NPs characterization using UV–visible spectrophotometer is considered the most widespread technique [28, 35]. In this study, UV–Vis spectrophotometer analyses were carried out at an absorbance spectrum range from 300 to 550 nm for that Ag-NPs that were synthesized extracellularly under light conditions.

**Figure 5:**
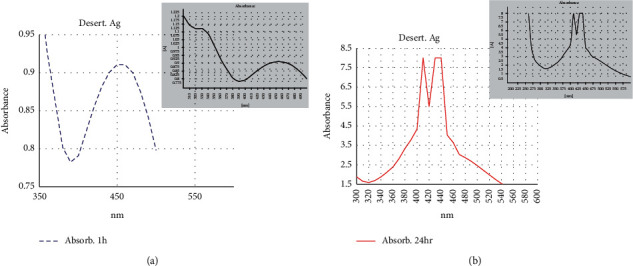
UV–Vis absorption spectra of Ag-NPs synthesized extracellularly by *Desertifilum tharense* after 1 h (a) and after 24 h (b).

**Figure 6:**
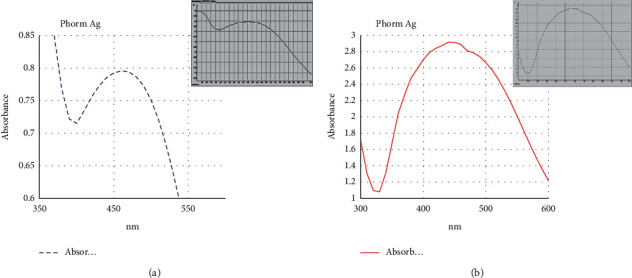
UV-visible absorption spectra of Ag-NPs synthesized extracellularly by *Phormidium ambiguum* after 1 h (a) and after 24 h (b).

For *Desertifilum tharense*, Ag-NPs gave the sharp SPR (surface plasmon resonance) peak at 450 nm after one hour (Figure 5(a)). With increasing reaction time reaching 24h, the peak shifted to a shorter wavelength region as two peaks appear at 410 and 435 nm (Figure 5(b)). Kathiraven *et al*. [36] reported that when the SPR band moved toward a shorter wavelength, it meant there was a decrease in particle size. This phenomenon was not observed for Ag-NPs synthesized by *Phormidium ambiguum* as the SPR peak appeared at 450 nm after 1 and 24 hours (Figure 6(a) and 6(b)). Ag-NPs give the plasmon resonance peak in the absorption spectrum between 410 and 450 nm [11]. Consequently, the results confirmed successful extracellular Ag-NPs synthesis by *Desertifilum tharense* and *Phormidium ambiguum* under light conditions.

#### 3.3.3. Scanning and Transmission Electron Microscopy

**Figure 7:**
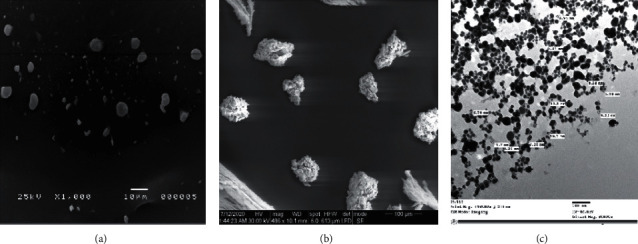
SEM micrographs of suspended Ag-NPs (a) and dried Ag-NPs (b) obtained from *Desertifilum tharense*. TEM micrographs of Ag-NPs (c) showed the spherical shape of Ag-NPs with a size of 6.24 nm to 11.7 nm.

**Figure 8:**
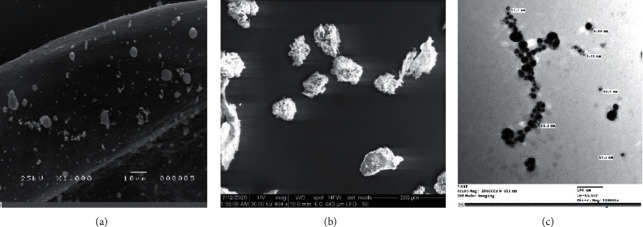
SEM micrographs of suspended Ag-NPs (a) and powder Ag-NPs (b) obtained from *Phormidium ambiguum*. TEM micrographs of Ag-NPs (c) showed the spherical shape of Ag-NPs with a size of 6.46–12.2 nm.

Scanning electron microscopy (SEM) micrographs indicated the presence of Ag-NPs produced by *Desertifilum tharense* (Figures 7(a) and 7(b)) and *Phormidium ambiguum* (Figures 8(a) and 8(b)) as irregular polydisperse clusters of particles.

The shape and size of Ag-NPs are determined by transmission electron microscopy (TEM). The TEM micrographs (7C) and (8C) showed the spherical shape of Ag-NPs produced by *Desertifilum tharense* and *Phormidium ambiguum*, respectively, and particles' dispersal without agglomeration. Also, TEM micrographs displayed the size of particles in a range of 6.24 nm to 11.7 nm and 6.46 nm to 12.2 nm for Ag-NPs produced by *Desertifilum tharense* and *Phormidium ambiguum*, correspondingly. The SPR peak at 435 nm of Ag-NPs synthesized by *Desertifilum tharense* confirmed the spherical shape of these particles reported by Govindaraju *et al*. [34].

Generally, cyanobacterial silver nanoparticles appear in different shapes. Patel *et al*. [2] studied the morphology of Ag-NPs produced by different cyanobacterial strains. They found that the Ag-NPs produced by *Anabaena* sp. And *Limnothrix* sp. appeared as elongated particles, while Ag-NPs of *Coelastrum* sp. And *Botryococcus braunii* appeared spherical. The particles formed by *Synechocystis* sp. were irregular clusters.

The size of Ag-NPs produced by *Desertifilum tharense* and *Phormidium ambiguum* was in a range reported by Singh *et al*. [37]. In other studies, the size of particles produced by cyanobacteria was larger. Rashed *et al*. [38] produced Ag-NPs with an average size of 60 nm from *Convolvulus arvensis* extract. The average size of Ag-NPs from *Chlorella* sp. was 90.6 nm [11]. Some cyanobacterial cultures synthesized silver particles with a size larger than 100 nm. Keskin *et al*. [12] and Kashyap *et al*. [11] produced particles with an average size of 140 nm from *Synechococcus* sp., and sizes of 136.2 and 241.8 nm from *Scenedesmus vacuolatus* and *Lyngbya putealis*, respectively.

#### 3.3.4. X-Ray Diffraction (XRD) Analysis

X-ray diffraction analysis (XRD) is a technique used to determine the crystallographic structure of a material by irradiating it with X-rays and measuring the intensities and scattering angles of the X-rays that leave the material. XRD pattern was investigated in the range of 0° to 80° at a diffraction angle of Pos (°2Th.).

**Figure 9:**
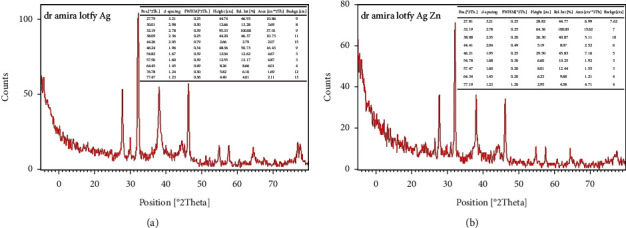
XRD pattern of Ag-NPs synthesized extracellularly by *Desertifilum tharense* (a) and *Phormidium ambiguum* (b).

The diffractogram of *Desertifilum tharense* Ag-NPs (Figure 9(a)) showed five main peaks of 32.19°, 38.089°, 44.2567°, 64.4547°, and 77.4727° corresponding to (101), (111), (200), (220), and (311) lattice planes, respectively, which were indexed in Joint Committee on Powder Diffraction Standards (JCPDS) file no. 84–0713 and 04–0783 as reported by Mahiuddin *et al*. [39]. Similar diffraction peaks were identified for *Phormidium ambiguum* Ag-NPs (Figure 9(b)).

The results of the XRD analysis confirmed that the biosynthesized Ag-NPs have a face-centered cubic (fcc) structure.

It was noticed that there were additional peaks at 54.82° and 57.5° in the diffractogram of *Desertifilum tharense* Ag-NPs. They were noticed at 54.78° and 57.47° in *Phormidium ambiguum* Ag-NPs. These peaks could be attributed to the presence of unreduced AgNO_3_, as stated by Mehta *et al*. [40]

#### 3.3.5. EDX Analysis

**Figure 10:**
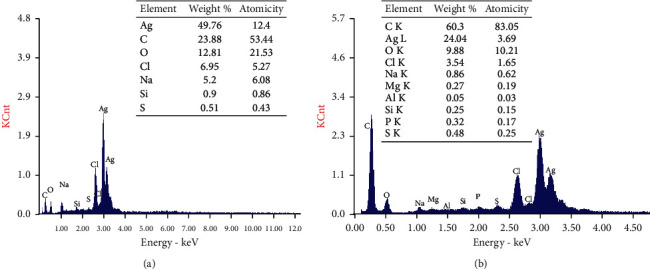
EDX quantitative analysis and spectra of Ag-NPs synthesized by *Desertifilum tharense* (a) and *Phormidium ambiguum* (b).

The purity, elemental constituents, and relative abundance of Ag-NPs synthesized by *Desertifilum tharense* and *Phormidium ambiguum* were analyzed employing Energy Dispersive X-ray (EDX) as presented in figures 10(a) and (10(b), respectively.

In figures (10(a) and 10(b), two peaks were observed at 3.0 and 3.2 keV for silver nanoparticles, revealing the presence of pure metallic Ag-NPs [2, 41].

In *Desertifilum tharense* Ag-NPs, EDX analysis (Figure 10(a)) suggested that the powder had high purity and intensity as the two emission peaks of metal silver showed more than 49%. The percentage relative composition of elements was carbon (C) 23.88%, oxygen (O) 12.81%, chlorine (Cl) 6.95%, sodium (Na) 5.2%, silicon (Si) 0.9%, and sulfur (S) 0.51%. While in the case of *Phormidium ambiguum*, EDX characterization (Fig.10B) exhibited less purity and weight as the two emission peaks of metal silver represent only more than 24.04%. The percentage relative composition of elements was carbon (C) 60.3%, oxygen (O) 9.88%, chlorine (Cl) 3.54%, sodium (Na) 0.86%, magnesium (Mg) 0.27%, ammonium (Al) 0.05%, silicon (Si) 0.25%, phosphorus (P) 0.32%, and sulfur (S) 0.48%.

XRD and EDX results confirm the successful synthesis of Ag-NPs in their oxide form or chloride form.

#### 3.3.6. Fourier Transform Infrared Spectroscopy (FTIR) Analysis

The structure of organic compounds on the surface of Ag-NPs was investigated using FTIR Spectroscopy.

**Figure 11:**
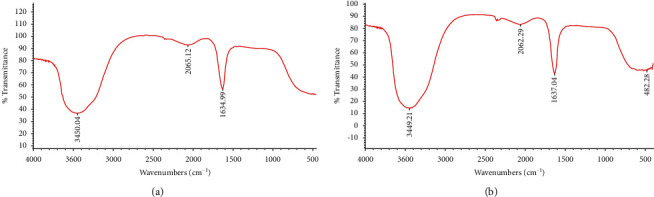
FTIR spectra of Ag-NPs synthesized extracellularly from *Desertifilum tharense* (a) and *Phormidium ambiguum* (b).

The FTIR spectrum of Ag-NPs synthesized by *Desertifilum tharense* and *Phormidium ambiguum* specified peaks around 3450, 2065, 1634, and 428 cm^−1^ (Figure 11). The strong absorption peak at 3450 cm^−1^ assigned to the O–H stretching vibration of polysaccharides and the N–H stretching vibration of proteins. It indicates that the polysaccharides and proteins found in the extracellular extract may play a role in the synthesis of Ag-NPs [26, 39]. Also, the polysaccharides and proteins, as organic capping agents, may contribute to particles stabilization. Jena *et al*. [29] reported that the capping peptide can bind to nanoparticles by free amino groups or by different residues inside the proteins. The peak around 1634 cm^−1^ attributed to stretching vibration of *C*=*O* in amides [39]. The peak at 428 cm^−1^ is related to binding Ag-NPs with oxygen from hydroxyl groups [42].

In addition to flavonoids, which have been assumed to act as both reducing and capping agents, cyanobacteria components used in this study contain a high amount of metabolites composed of aromatic rings with reactive –OH groups (Figure 11), which have been presumed to act as both reducing and capping agents. Flavonoids, particularly the –OH groups present in flavonoids, are responsible for the reduction of silver ions by the release of hydrogen ions during the tautomeric transformation, which had also been proposed by [5].

### 3.4. Antibacterial Activity

Currently, using nanoparticles as antimicrobial agents is gaining excessive interest. Generally, the nanoparticles are evaluated as antimicrobials alone or in combination with antibiotics. The nanoparticles can increase the antimicrobial effect of some antibiotics against Gram-positive and Gram-negative bacteria [43].

In this study, the efficiency of extracellular Ag-NPs synthesized from two cyanobacterial strains was measured against six pathogenic bacterial strains. The results were compared with vancomycin hydrochloride (100 mg/ml) and AgNO_3_ (1 mM) for evaluating the relative percentage inhibition of Ag-NPs.

*Desertifilum tharense* Ag-NPs displayed the largest inhibition zone ranging from 9 mm against *Micrococcus luteus* ATCC 10240 to 25 mm against methicillin-resistant *S*. *aureus* (MRSA) ATCC 43300 (Table 1). The greatest relative percentage inhibition was recorded against *S. typhimurium* ATCC 14028 (129.6%), followed by MRSA ATCC 43300 and *E*. *coli* O157 : H7 wild type strain 93111 (100%).

For *Phormidium ambiguum* Ag-NPs, the inhibition zone diameter was in a range of 9 mm to 18 mm. The maximum relative percentage inhibition of 86.6% was displayed against *Salmonella typhimurium* ATCC 14028, followed by *E*. *coli* O157 : H7 wild type strain 93111 (81.7%) and *P*. *aeruginosa* ATCC 9027 (75.1%). 

The great antibacterial activity of *Desertifilum tharense* Ag-NPs against tested pathogenic bacteria could be attributed to their particle size (6.24 nm to 11.7 nm), which was smaller than that of *Phormidium ambiguum* Ag-NPs (6.46 nm to 12.2 nm). It is in accord with the findings that belong to Ivask *et al*. [44]. Patel *et al*., [2] found that the antibacterial activity of Ag-NPs was inversely proportional with their particle size as a smaller size meant a larger surface area, which facilitated the interaction between nanoparticles and microbial cells. The particles with the size of 10 nm or smaller can easily penetrate the bacterial cell wall and interact with the cytosol biomolecules. We knew that the antibacterial activity of Ag-NPs was attributed not only to the small size but also to their positive charge. Generally, particles had electrostatically positive charges, bind to lipopolysaccharides on the outer membrane of Gram-bacteria, or bind to lipoteichoic acids on the surfaces of Gram + bacteria. It led to the facilitation of cell penetration [45]. Rajeshkumar *et al*. [3] and Otari *et al*. [46] found that a spherical shape with a small size of Ag-NPs increases the contact area, ensuring the elimination of bacterial growth.

Silver is a poisonous but precious heavy metal that was treated as a noble metal in the comprehensive ancient Indian medical text, the great “Charaka Samhita” [5],and it has widespread applications in various biomedical and environmental divisions, especially in health, wound dressings, medical supplements, and industrial products for human utilization, to inhibit pathogen growth. It is incorporated in cosmetics as an antiseptic, and it is used in medical textiles to eliminate microbes from the clinical environment [8]. Also, it is used in feed supplementation [5]. The wide usage of the metal has made strong environmental apprehensions. Henceforth, there is a cumulative call for the progress of a modest, low-cost, and ecological method for the remediation of silver [47].

Metal nanoparticles are currently synthesized via physical and chemical processes. As traditional manufacturing methods are typically costly, labor-intensive, and produce hazardous byproducts that are harmful to the environment and living organisms [8], there is a pressing need to develop ecofriendly, safe, dependable, and clean nanoparticle preparation methods. Various biological approaches, such as the utilization of the plant *Streptomyces*, extracts, fungus, bacteria, and algae, are regarded as safe and nontoxic, and they provide a more environmentally-sound synthesis of nanoparticles.

Despite the fact that numerous metallic nanoparticles have been manufactured using biological sources, such as plants, microbes, algae, and fungus, the specific method of synthesis remains unknown in the case of Ag-NPs. However, it has been suggested that nanoparticle synthesis is divided into three steps: (1) metal ion reduction, (2) clustering, and (3) nanoparticle production. Reactions in each of these steps directly pertain to the concentration, temperature, pH, and composition of the biological material, and the metal salt concentrate, as mentioned by [5]. In the aqueous environment, AgNO_3_ molecules dissociate into silver ions (Ag+) and nitrate ions (NO_3_). The reduction of two silver ions, specifically the –OH groups, occurs when these two protons are released from flavonoid molecules, resulting in the creation of silver nanoparticles [5].

The following are the proposed mechanisms for Ag-NPs' mode of action: the reduction of Ag + ions occurs extracellularly by reductase enzymes and electron shuttle quinones. In the presence of enzymes like NADH-dependent reductases, silver ions are reduced intracellularly by electrons produced by the organisms to avoid destruction [8]. When silver ions produced from silver nanoparticles come into contact with bacteria, they may inhibit the production of some enzymes and cellular proteins required for adenosine triphosphate (ATP) synthesis or have an impact on bacterial DNA replication. In other pieces of research, silver ions may disrupt the working of the membrane-bound enzymes of the respiratory chain. Other studies showed that the bacteria cause damage by rupturing the plasma membrane or by blocking respiration in association with oxygen and sulfhydryl (SH) groups on the cell wall to form *R* S S *R* bonds, thus leading to the exhaustion of intercellular ATP.

Generally, silver nanoparticles are synthesized through two pathways, comprising enzymatic and nonenzymatic reductions [46]. The nitrate reductase enzyme, which is involved in the enzymatic synthesis of silver nanoparticles, converts nitrate to nitrite. In addition, an electron shuttle is induced, leading to the reduction of silver ions to silver nanoparticles. The enzymatic production of silver nanoparticles is a fast and nontoxic approach. Conversely, the nonenzymatic synthesis of Ag-NPs depends on the chemical reduction of silver atoms using reducing and stabilizing compounds produced by plants or microorganisms.

The mode of action of Ag-NPs as antibacterial mainly includes the loss of the outer membrane integrity in G-bacteria. The permeabilization of the bacterial cell membrane by the formation of pores across it resulted in the release of the cellular material, causing cell death [48, 49]. On the other hand, the pores formed in the cell membrane facilitate the inflow of Ag-NPs into the cell to combine with proteins containing sulfur and DNA, which caused damage to the DNA and enzymes, leading to blocking vital metabolic processes [50].

The effectiveness of Ag-NPs as antibacterial is determined by their size, surface functionalization, surface area in contact with microbial cells, concentration [8], microbial source, pH, incubation temperature, and whether the bacterial reduction occurs intracellularly or extracellularly [51].

### 3.5. Free Radical Scavenging Potential of Ag-NPs

Results showed that the cyanobacterial Ag-NPs have the highest scavenging activity when compared with the cyanobacterial extracellular extract. Silver nanoparticles of *Phormidium ambiguum* exhibited the highest scavenging activity of 48.7% when compared with that of the silver nanoparticles of *Desertifilum tharense,* which displayed 43.753% (Table 2).

These results agree well with the previous reports on the antioxidant property of the biofabricated Ag-NPs using extracts, including plant extracts [52], bacteria [47], cyanobacteria [53], and microalgae [54]. The antioxidant characteristics of nanoparticles are mostly determined by the methods used in nanoparticle preparation and the ability of the living organism to adapt to harsh environmental conditions, such as cyanobacteria's phototrophic existence and constant exposure to high oxygen and radical stressors. As a result, cyanobacteria have a great capacity for producing a variety of effective anti-oxidant and anti-radical compounds. Cyanobacteria are photo-autotrophs, and they require a lot of light to create their food. Furthermore, cyanobacteria are creatures that fix nitrogen gas, and because nitrogen fixation is an obligatory anaerobic process, oxygen poses a threat to these species [55–57].

As mentioned by [47], antioxidants protect the cells from free radical damage. An antioxidant prevents oxidation by neutralizing the generated free radicals, which causes the antioxidant to oxidize. DPPH is a purple-colored organic radical that is commercially accessible. The purple tint is diminished with reduction, indicating that the substance added had antioxidant activity. The percentage of DPPH left showed the DPPH free radical scavenging activity. Even after 30 minutes, there was still a lot of activity (94.01 percent).

## 4. Conclusion

In this study, Egyptian cyanobacterial isolates of *Phormidium ambiguum* and a novel *Desertifilum tharense* were found to be potent for the green synthesis of Ag-NPs by the biological reduction of Ag ions into NPs extracellularly and intracellularly under light and dark conditions. The best results were observed using culture supernatants, especially under light conditions, because of microbial products that facilitate this reaction extracellularly. Therefore, further characterization tests were needed to assess the antibacterial and antioxidant activities of Ag-NPs synthesized extracellularly under light conditions.

These cyanobacteria can produce smaller spherical particles with face-centered cubic structures. The presence of amides and hydroxyl groups indicates that proteins and polysaccharides could be considered important factors in the biosynthesis of Ag-NPs. Generally, the characterization assay showed that novel *Desertifilum tharense* cyanobacteria have superior power in the green synthesis of Cyano-AgNPs. The current findings indicate that the biosynthesized Ag-NPs may be potent antibacterial agents against different pathogenic bacteria and could be used as alternatives to antibiotics. Further studies will recommend knowing the optimal conditions for Ag-NPs biosynthesis. Also, more biological characterizations and *in vivo* experiments are required to establish the real potential for their application in the medical and food sectors.

## Figures and Tables

**Table 1:** Antibacterial activity of Ag-NPs synthesized extracellularly from *Desertifilum tharense* and *Phormidium ambiguum* under light conditions.

Indicator microorganisms	*Desertifilum tharense* Ag-NPs		*Phormidium ambiguum* Ag-NPs	
	Inhibition zone (mm)	Relative percentage inhibition (%)	Inhibition zone (mm)	Relative percentage inhibition (%)
*B*. *cereus* ATCC 10876	12	84.8	9	58.2
MRSA ATCC 43300	25	100.0	17	44.8
*M*. *luteus* ATCC 10240	9	45.0	9	45.0
*S*. *typhimurium* ATCC 14028	17	129.6	13	86.6
*P*. *aeruginos* ATCC 9027	14	87.1	13	75.1
*E*. *coli* O157 : H7 wild type strain 93111	21	100.0	18	81.7

**Table 2:** Antioxidant capacity (%) of Ag-NPs synthesized extracellularly from *Desertifilum tharense* and *Phormidium ambiguum* under light conditions.

Tested cyanobacterial strains	Absorbance at 515 nm			DPPH scavenging effect (%)	
	Methanolic DPPH	Extracellular extract	Ag-NPs	Extracellular extract	Ag-NPs
*Desertifilum tharense*	0.770	0.492	0.433	36.143	43.753
*Phormidium ambiguum*	0.770	0.508	0.395	33.974	48.701

## Data Availability

The data used to support the findings of this study are included within the article.

## References

[1] Anbang W., Jingyang Li A. A., AL-Harbi M. S. (2021). Mechanisms of chitosan nanoparticles in the regulation of cold stress resistance in banana plants. *Nanomaterials*.

[2] Patel V., Berthold D., Puranik P., Gantar M. (2015). Screening of cyanobacteria and microalgae for their ability to synthesize silver nanoparticles with antibacterial activity. *Biotechnology Reports*.

[3] Rajeshkumar S., Malarkodi C., Paulkumar K., Vanaja M., Gnanajobitha G., Annadurai G. (2014). Algae mediated green fabrication of silver nanoparticles and examination of its antifungal activity against clinical pathogens. *International Journal of Metals*.

[4] Li S., Zhang T., Tang R., Qiu H., Wang C., Zhou Z. (2015). Solvothermal synthesis and characterization of monodisperse superparamagnetic iron oxide nanoparticles. *Journal of Magnetism and Magnetic Materials*.

[5] Some S., Bulut O., Biswas K. (2019). Effect of feed supplementation with biosynthesized silver nanoparticles using leaf extract of Morus indica L. V1 on *Bombyx mori* L. (Lepidoptera: bombycidae). *Scientific Reports*.

[6] Zak K., Ebrahimizadeh M., Abd-Majid W., Yousefi S. (2011). Effects of annealing temperature on some structural and optical properties of ZnO nanoparticles prepared by a modified sol–gel combustion method. *Ceramics International*.

[7] Al Rohily K., El-Hamshary H., Ghoneim A., Modaihsh A. (2020). Controlled Release of Phosphorus from Superabsorbent Phosphate-Bound Alginate-Graft-Polyacrylamide: Resistance to Soil Cations and Release Mechanism. *ACS Omega*.

[8] El-Naggar N. E. A., Hussein M. H., El-Sawah A. A. (2017). Bio-fabrication of silver nanoparticles by phycocyanin, characterization, in vitro anticancer activity against breast cancer cell line and in vivo cytotxicity. *Scientific Reports*.

[9] Alharbi K., Ghoneim A., Ebid A., El-Hamshary H., El-Newehy M. H. (2018). Controlled release of phosphorous fertilizer bound to carboxymethyl starch-g-polyacrylamide and maintaining a hydration level for the plant. *International Journal of Biological Macromolecules*.

[10] Ghoneim A. M., Elbassir O. I., Modahish A. S., Mahjoub M. O. (2017). Compost production from olive tree pruning wastes enriched with phosphate rock. *Compost Science & Utilization*.

[11] Kashyap M., Samadhiya K., Ghosh A., Anand V., Shirage P. M., Bala K. (2019). Screening of microalgae for biosynthesis and optimization of Ag/AgCl nano hybrids having antibacterial effect. *RSC Advances*.

[12] San Keskin N. O., Koçberber Kılıç N., Dönmez G., Tekinay T. (2016). Green synthesis of silver nanoparticles using cyanobacteria and evaluation of their photocatalytic and antimicrobial activity. *Journal of Nano Research*.

[13] Kumar D., Kaštánek P., Adhikary S. P. (2018). Exopolysaccharides from cyanobacteria and microalgae and their commercial application. *Current Science*.

[14] Malhotra A., Dolma K., Kaur N., Rathore Y., Mayilraj S., Choudhury A. (2013). Biosynthesis of gold and silver nanoparticles using a novel marine strain of Stenotrophomonas. *Bioresource Technology*.

[15] Ding Z., Majrashi M. A., Ghoneim A. M., Ali E. F., Eissa M. A. (2022). Irrigation and biochar effects on pearl millet and kinetics of ammonia volatilization from saline sandy soils. *Journal of Soil Science and Plant Nutrition*.

[16] Mahdieh M., Zolanvari A., Azimee A. S., Mahdieh M. (2012). Green biosynthesis of silver nanoparticles by Spirulina platensis. *Scientia Iranica*.

[17] Ninganagouda S., Rathod V., Singh D. (2014). Growth kinetics and mechanistic action of reactive oxygen species released by silver nanoparticles from Aspergillus Niger on of reactive oxygen species. *The Journal of Physical Chemistry B*.

[18] Beyth N., Yudovin-Farber I., Perez-Davidi M., Domb A. J., Weiss E. I. (2010). Polyethyleneimine nanoparticles incorporated into resin composite cause cell death and trigger biofilm stress in vivo. *Proceedings of the National Academy of SciencesProceedings of the National Academy of Sciences*.

[19] Choi O., Deng K. K., Kim N.-J., Ross L., Surampalli R. Y., Hu Z. (2008). The inhibitory effects of silver nanoparticles, silver ions, and silver chloride colloids on microbial growth. *Water Research*.

[20] Rippka R., Deruelles J., Waterbury J. b, Herdman M., Stanier R. (1979). Generic assignments, strain histories and properties of pure cultures of cyanobacteria. *Journal of General MicrobioZogy*.

[21] Waterbury J. B. (2006). The cyanobacteria—isolation, purification and identification. *Prokaryotes*.

[22] Koo H., Mojib N., Hakim J. (2019). Bacteria (16S) in growth laminae of a large conical mats from Lake Untersee, East Antarctic.

[23] Saitou N., Nei M. (1987). The neighbor-joining method: a new method for reconstructing phylogenetic trees. *Molecular Biology and Evolution*.

[24] Tamura K., Nei M., Kumar S. (2004). Prospects for inferring very large phylogenies by using the neighbor-joining method.no title. 101:11030-11035. *Proceedings of the National Academy of Sciences*.

[25] Mahamuni P. P., Patil P. M., Dhanavade M. J., Badiger M. V. (2018). Synthesis and characterization of zinc oxide nanoparticles by using polyol chemistry for their antimicrobial and antibiofilm activity. *Biochemistry and Biophysics Reports*.

[26] Hamida R. S., Abdelmeguid N. E., Ali M. A., Bin-Meferij M. M., Khalil M. I. (2020). Synthesis of silver nano using a novel cyano desertifilum, antibacterial and cytotoxicity effects, 15. *International Journal of Nanomedicine*.

[27] Sadowski Z. (2010). Biosynthesis and application of silver and gold nanoparticles. *Silver Nanoparticles*.

[28] Gowramma B., Keerthi U., Rafi M., Muralidhara Rao D. (2015). Biogenic silver nanoparticles production and characterization from native stain of Corynebacterium species and its antimicrobial activity. *3 Biotech*.

[29] Jena J., Pradhan N., Prasad Dash B., Behari Sukla L., kumar Panda Affiliations P. (2013). Biosynthesis and characterization of silver nanoparticles using microalga Chlorococcum humicola and its antibacterial activity. *International Journal of Nano and Biomaterials*.

[30] Kumar G., Karthik L., Bhaskara Rao K. V. (2010). Antibacterial activity of aqueous extract of Calotropis gigantea leaves - an in vitro study. *International Journal of Pharmaceutical Sciences Review and Research*.

[31] Hamouda R. A., Al-Saman M. A., El-Sabbagh S. M., C G. W. A. E.-S., Hendawy A. N. (2017). Approach to improve bioactive compounds of cyano anabaena oryzae using factorial design.

[32] Khan M. J., Shameli K., Sazili A. Q., Selamat J., Kumari S. (2019). Rapid green synthesis and characterization of silver nanoparticles arbitrated by curcumin in an alkaline medium. *Molecules (Basel, Switzerland)*.

[33] Saifuddin N., Wong C., Yasumira A. N. (2009). Rapid biosynthesis of silver nanoparticles using culture supernatant of bacteria with microwave irradiation. *E-J Chem*.

[34] Govindaraju K., Kiruthiga V., Kumar V. G., Singaravelu G. (2009). Extracellular synthesis of silver nanoparticles by a marine alga, Sargassum wightii grevilli and their Antibacterial effects. *Journal of Nanoscience and Nanotechnology*.

[35] Sun Y., Atorngitjawat P., Meziani M. (2001). Preparation of silver nanoparticles via rapid expansion of water in carbon dioxide microemulsion into reductant solution. *Langmuir*.

[36] Kathiraven T., Sundaramanickam A., Shanmugam N., Balasubramanian T. (2014). Green synthesis of silver nanoparticles using marine algae Caulerpa racemosa and their antibacterial activity against some human pathogens. *Applied Nanoscience*.

[37] Singh G., Babele P. K., Shahi S. K., Sinha R. P., Tyagi M. B., Kumar A. (2014). Green synthesis of silver nanoparticles using cell extracts of Anabaena doliolum and screening of its antibacterial and antitumor activity. *Journal of Microbiology and Biotechnology*.

[38] Rashed S. Al, Shehri S. Al, Moubayed N. M. S. (2018). Extracellular biosynthesis of silver nanoparticles from Cyanobacteria. *Biomedical Research*.

[39] Mahiuddin M. D., Saha P., Ochiai B. (2020). Green synthesis and catalytic activity of silver nanoparticles based on Piper chaba stem extracts. *Nanomaterials*.

[40] Mehta B. K., Chhajlani M., Shrivastava B. D. (2017). Green synthesis of silver nanoparticles and their characterization by XRD. *IOP Conf. Series: Journal of Physics: Conference Series*.

[41] Magudapathy P., Gangopadhyay P., Panigrahi B. K., Nair K. G. M., Dhara S. (2001). Electrical transport studies of Ag nanoclusters embedded in glass matrix. *Physica B: Condensed Matter*.

[42] Al-Katib M., Al-Shahri Y., AL-Niemi A. (2015). Biosynthesis of silver nanoparticles by Cyanobacterium Gloeocapsa sp. *International Journal of Enhanced Research in Science, Technology & Engineering*.

[43] Li X., Xu H., Chen Z.-S., Chen G. (2011). Biosynthesis of nanoparticles by microorganisms and their applications. *Journal of Nanomaterials*.

[44] Ivask A., Kurvet I., Kasemets K. (2014). Size-dependent toxicity of silver nanoparticles to bacteria, yeast, algae, crustaceans and mammalian cells in vitro. *PLoS One*.

[45] Glinel K, Thebault P, Humblot V, Pradier CM, Jouenne T (2012). Antibacterial surfaces developed from bio-inspired approaches. *Acta Biomaterialia*.

[46] Otari S. V., Patil R. M., Ghosh S. J., Thorat N. D., Pawar S. H. (2015). Intracellular synthesis of silver nanoparticle by actinobacteria and its antimicrobial activity. *Spectrochimica Acta Part A: Molecular and Biomolecular Spectroscopy*.

[47] Ibrahim S., Ahmad Z., Manzoor M. Z., Mujahid M., Faheem Z., Adnan A. (2021). Optimization for biogenic microbial synthesis of silver nanoparticles through response surface methodology, characterization, their antimicrobial, antioxidant, and catalytic potential. *Scientific Reports*.

[48] Dehkordi S. H., Hosseinpour F., Kahrizang A. E. (2011). An in vitro evaluation of antibacterial effect of silver nanoparticles on *Staphylococcus aureus* isolated from bovine subclinical mastitis. *African Journal of Biotechnology*.

[49] Raffi M., Hussain F., Bhatti T. M., Akhter J. I., Hameed A., Hasan M. M. (2008). Antibacterial characterization of silver nanoparticles against *E. coli* ATCC-15224. *Journal of Materials Science and Technology*.

[50] Hamouda R. A., Hussein M. H., Abo-elmagd R. A., Bawazir S. S. (2019). Synthesis and biological characterization of silver nanoparticles derived from the cyanobacterium Oscillatoria limnetica. *Scientific Reports*.

[51] Peiris M. M. K., Fernando S. S. N., Jayaweera P. M., Arachchi N. D. H., Guansekara T. D. C. P. (2018). Comparison of antimicrobial properties of silver nanoparticles synthesized from selected bacteria. *Indian Journal of Microbiology*.

[52] Anjana V. N., Joseph M., Francis S., Joseph A., Koshy E. P., Mathew B. (2021). Microwave assisted green synthesis of silver nanoparticles for optical, catalytic, biological and electrochemical applications. *Artificial Cells, Nanomedicine, and Biotechnology*.

[53] Hassouani M., Sabour B., Belattmania Z. (2017). In vitro anticancer, antioxidant and antimicrobial potential of Lyngbya aestuarii (Cyanobacteria) from the Atlantic coast of Morocco. *Journal of Materials and Environmental Science*.

[54] Jerez-Martel I., García-Poza S., Rodríguez-Martel G., Rico M., Afonso-Olivares C., Gómez-Pinchetti J. L. (2017). Phenolic profile and antioxidant activity of crude extracts from microalgae and cyanobacteria strains. *Journal of Food Quality*.

[55] Babić O., Kovač D., Rašeta M., Šibul F., Svirčev Z., Simeunović J. (2016). Evaluation of antioxidant activity and phenolic profile of filamentous terrestrial cyanobacterial strains isolated from forest ecosystem. *Journal of Applied Phycology*.

[56] Hanna A. L., Hamouda H., Goda H., Elsayed T., Sadik M. (2021). Biosynthesis and Characterization of Silver Nanoparticles produced by Phormidium ambiguum and Desertifilum tharense. *A Preprint Has Previously Been Published in Research Square*.

[57] Ilyas F., Ali M., Modhish A. Synchronisation of zinc application rates with arbuscular mycorrhizal fungi and phosphorus to maximise wheat growth and yield in zinc-deficient soil. *Crop & Pasture Science*.

